# Supplementary material for: Splice-Junction-Based Mapping of Alternative Isoforms in the Human Proteome
Source: Cell Rep. Author manuscript; Available in PMC 2020 Jan 15. (PMC6961840; doi:10.1016/j.celrep.2019.11.026)
Supplement: 3 [file NIHMS1546469-supplement-3.zip › DF2/PXD000561/Pancreas-61-P53396-AKPAMPQGK.pdf]

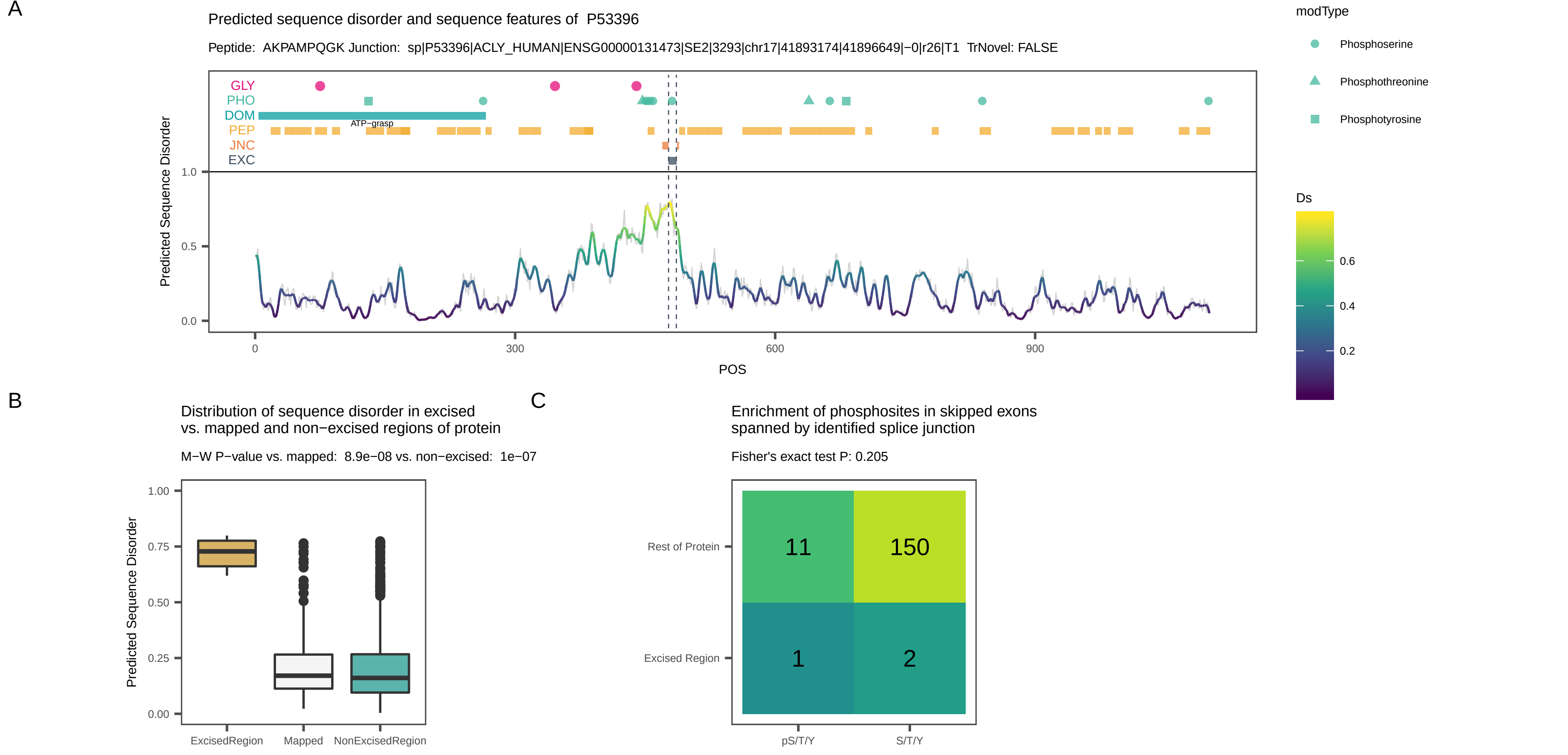

A
Predicted sequence disorder and sequence features of P53396
Peptide: AKPAMPQGK Junction: sp|P53396|ACLY_HUMAN|ENSG00000131473|SE2|3293|chr17|41893174|41896649|−0|r26|T1 TrNovel: FALSE
GLY
PHO
DOM
PEP
JNC
EXC
ATP−grasp
Predicted Sequence Disorder
1.0
0.5
0.0
0
300
600
900
POS
modType
Phosphoserine
Phosphothreonine
Phosphotyrosine
Ds
0.6
0.4
0.2
B
Distribution of sequence disorder in excised vs. mapped and non−excised regions of protein
M−W P−value vs. mapped: 8.9e−08 vs. non−excised: 1e−07
Predicted Sequence Disorder
1.00
0.75
0.50
0.25
0.00
ExcisedRegion
Mapped
NonExcisedRegion
C
Enrichment of phosphosites in skipped exons spanned by identified splice junction
Fisher's exact test P: 0.205
Rest of Protein
Excised Region
11
150
1
2
pS/T/Y
S/T/Y
